# Characterization of the complete chloroplast genome of the Musk Larkspur *Delphinium brunonianum* (Ranunculales: Ranunculaceae)

**DOI:** 10.1080/23802359.2020.1775522

**Published:** 2020-06-08

**Authors:** Qien Li, Xiao Guo, Farong Yuan, Cairang Nima, Duojie Dongzhi, Duojie, XianJia Li

**Affiliations:** a Tibetan Medicine Research Center, Tibetan Medical College, Qinghai University, Xining, People’s Republic of China; b State Key Laboratory of Tibetan Medicine Research and Development, Qinghai University, Xining, People’s Republic of China; c Jinhe Tibetan Medicine Co., Ltd, Xining, People’s Republic of China

**Keywords:** Iterative mapping, Musk Larkspur, *Delphinium brunonianum*, phylogeny, plastid genome

## Abstract

Musk Larkspur (*Delphinium brunonianum*) is a perennial herb of the family Ranunculace with medicinal values. In this study, the chloroplast (cp) genome of this herb was determined to be 153,926 bp long with an A + T-biased base composition, and comprises a pair of inverted repeat (IR) regions (26,559 bp), separated by a large single-copy (LSC) region (84,512 bp) and a small single-copy (SSC) region (16,296 bp). A total of 112 gene species were annotated with 19 of them being completely or partially duplicated. Eighteen gene species harbor one or two introns. Phylogenetic analysis challenged the monophyly of the subfamily Ranunculoideae.

Musk Larkspur (*Delphinium brunonianum*) is a perennial herb of the family Ranunculace, and is native to southern Tibet of China, Afghanistan, Nepal and northern Pakistan, occurring in grassy or gravelly places with an elevation of 4500–6000 m (Wang and Warnock 2001). The dried aerial parts of this herb are used in traditional Tibetan medicine for the treatment of skin itching, various infectious diseases, influenza and snake bites, etc. (Yang 1991). Up to now, almost all studies of *D. brunonianum* have been restricted to its phytochemistry (Asif et al. 2019; Zou, Dawa, et al. 2019; Zou, Zeren, et al. 2019). Little is known about its genetics or genomics, although such a knowledge would be important to its conservation and sustainable exploitation. The present study for the first time presents its complete chloroplast (cp) genome, which is currently accessible from GenBank under the accession number MT457851.

The leaf tissues used for DNA extraction in this study were collected from a single individual of *D. brunonianum* in Namling County, Shigatse City (89°47′19″E, 29°63′22″N) with the voucher specimen held in (accession number: LQE-2019-067). The high-throughput DNA sequencing was performed on the Illumina HiSeq X Ten Sequencing System (Illumina, CA, USA) by Novogene Biotech Co., Ltd. (Beijing, China). In all, 4.92 M of 150-bp raw reads were used for the assembly of cp genome using MITObim v1.9 (Hahn et al. 2013) with that of *Delphinium ceratophorum* (MK253460) (He et al. 2019) as the initial reference. The resultant cp genome sequence was annotated in Geneious Prime 2020 (Biomatters Ltd., Auckland, New Zealand) by comparing with those of its consubfamilial counterparts, e.g., *Consolida ajacis* (MK569484) (Zhai et al. 2019), *Delphinium maackianum* (MN648402) (Park et al. 2020), *Delphinium anthriscifolium* (MK253461) and *Delphinium ceratophorum* (MK253460) (He et al. 2019).

The cp genome of *D. brunonianum* is a double-stranded circular molecule of 153,926 bp in size, and comprises a pair of inverted repeat (IR) regions (26,559 bp), separated by a large single-copy (LSC) region (84,512 bp) and a small single-copy (SSC) region (16,296 bp). It has an A + T-biased base composition with an overall A + T content of 61.7% (‘light strand’). A total of 112 gene species were annotated, including 78 protein-coding (PCG), 30 tRNA and four rRNA gene species. Partial or complete gene duplication was detected in 19 gene species, including eight PCGs, seven tRNAs and all four rRNAs. Furthermore, a single intron is present in ten PCG species (*atpF*, *ndhA*, *ndhB*, *petB*, *petD*, *rpl2*, *rpl16*, *rpoC1*, *rps12* & *rps16*) and six tRNA gene species (*trnA-UGC*, *trnG-GCC*, *trnI-GAU*, *trnK-UUU*, *trnL-UAA* & *trnV-UAC*), and a couple of introns in two PCG species (*clpP* & *ycf3*).

A phylogeny of the family Ranunculaceae was reconstructed based on the Bayesian analysis of concatenated cp protein-coding sequences with MrBayes v3.1.1 (Huelsenbeck and Ronquist 2001; Ronquist and Huelsenbeck 2003) as in TOPALi v2.5 (Milne et al. 2009) (Figure 1). ‘GTR + G + I’ was selected as the best-fit nucleotide substitution model. The outgroup taxa included in the phylogenetic analysis are three species of the family Berberidaceae, including *Berberis ganpinensis* (MN417307) (Huang et al. 2019), *Caulophyllum robustum* (MH423066) (Sun et al. 2018) and *Gymnospermium kiangnanense* (MH298010) (Yang et al. 2018). The monophyly of the ten tribes within the subfamily Ranunculoideae is well supported by the present study. However, in accordance with previous reports (Wang et al. 2009; Cossard et al. 2016), our result also challenged the monophyly of Ranunculoideae due to the position of the tribe Adonideae being sister to the subfamily Thalictroideae. In addition, *D. brunonianum* was found to be most closely related to two of its congeners, i.e., *D. ceratophorum* (MK253460) and *D. maackianum* (MN648402).

**Figure 1.**
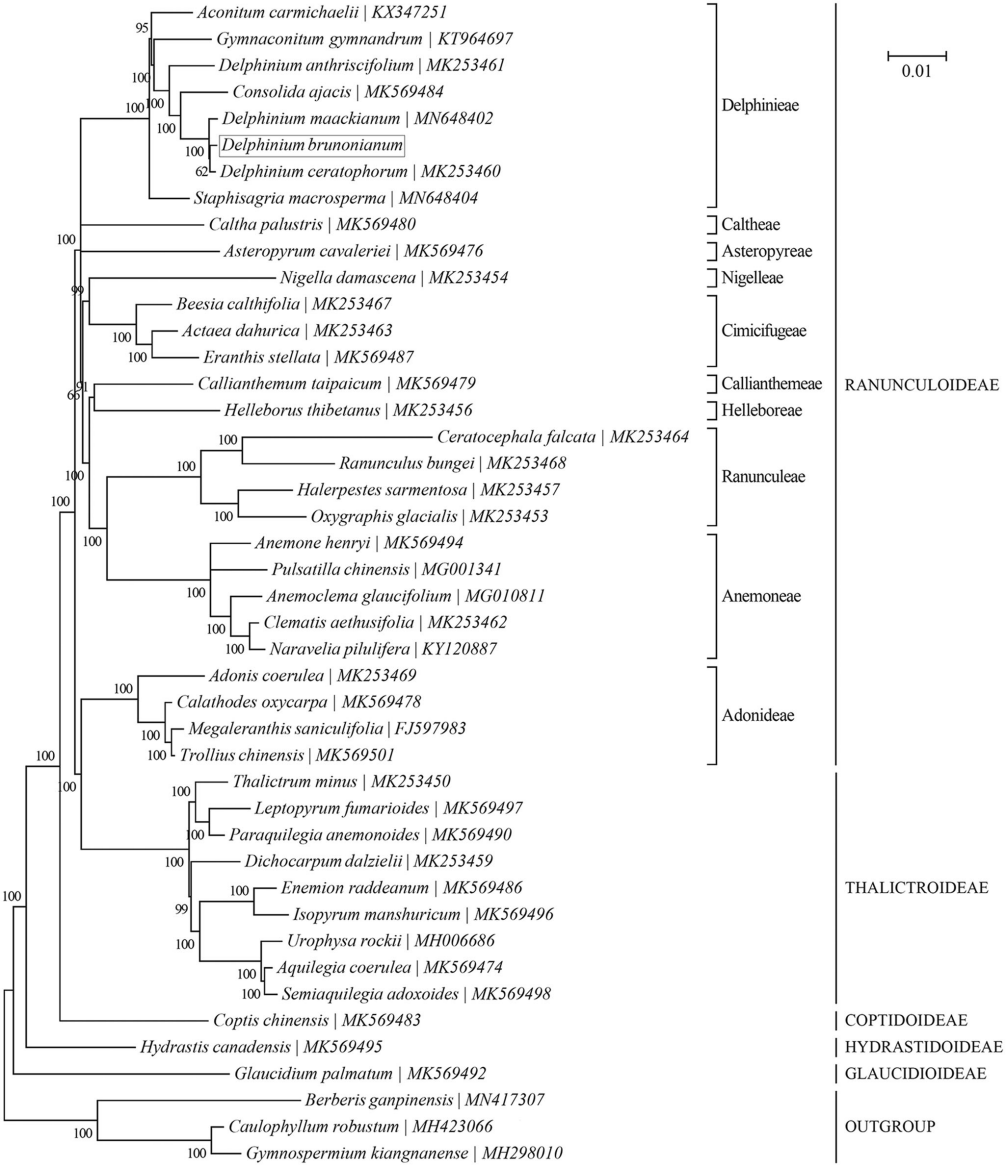
Phylogenetic relationships of 41 species within the family Ranunculaceae based on the Bayesian analysis of the concatenated coding sequences of chloroplast PCGs. The best-fit nucleotide substitution model is ‘GTR + G+I’. Tribe-level (specifically for the subfamily Ranunculoideae) and subfamily-level taxonomy is presented for each taxon. Three species within the family Berberidaceae were included as outgroup taxa.

## Data Availability

The data that support the findings of this study are openly available in GenBank of NCBI at https://www.ncbi.nlm.nih.gov, reference number MT457851.
